# Genetic and pharmacological inhibition of XBP1 protects against APAP hepatotoxicity through the activation of autophagy

**DOI:** 10.1038/s41419-022-04580-8

**Published:** 2022-02-10

**Authors:** Hui Ye, Chaobo Chen, Hanghang Wu, Kang Zheng, Beatriz Martín-Adrados, Esther Caparros, Rubén Francés, Leonard J. Nelson, Manuel Gómez del Moral, Iris Asensio, Javier Vaquero, Rafael Bañares, Matías A. Ávila, Raúl J. Andrade, M. Isabel Lucena, Maria Luz Martínez-Chantar, Helen L. Reeves, Steven Masson, Richard S. Blumberg, Jordi Gracia-Sancho, Yulia A. Nevzorova, Eduardo Martínez-Naves, Francisco Javier Cubero

**Affiliations:** 1 Department of Immunology, Ophthalmology and ENT, Complutense University School of Medicine, 28040 Madrid, Spain; 2 12 de Octubre Health Research Institute (imas12), 28007 Madrid, Spain; 3 Department of Anesthesiology, ZhongDa Hospital Southeast University, 210009 Nanjing, China; 4 Department of General Surgery, Wuxi Xishan People’s hospital, 214105 Wuxi, China; 5 Department of Hepatic-Biliary-Pancreatic Surgery, the Affiliated Drum Tower Hospital of Nanjing University Medical school, 210000 Nanjing, China; 6 Departmento de Medicina Clínica, Universidad Miguel Hernández, 03550 San Juan de Alicante, Spain; 7 Instituto ISABIAL-FISABIO, Hospital General Universitario de Alicante, 03010 Alicante, Spain; 8 Centro de Investigación Biomédica en Red de Enfermedades Hepáticas y Digestivas (CIBEREHD), 28029 Madrid, Spain; 9 Institute for Bioengineering (IBioE), Human Tissue Engineering, Faraday Building, The University of Edinburgh, EH9 3DW Edinburgh, Scotland UK; 10 Department of Cell Biology, Complutense University School of Medicine, 28040 Madrid, Spain; 11 Servicio de Aparato Digestivo, Hospital General Universitario Gregorio Marañón, 28007 Madrid, Spain; 12 Instituto de Investigación Sanitaria Gregorio Marañón (IiSGM), 28007 Madrid, Spain; 13 Hepatology Program, CIMA, University of Navarra, 31008 Pamplona, Spain; 14 Instituto de Investigaciones Sanitarias de Navarra IdiSNA, 31008 Pamplona, Spain; 15 Unidad de Gestión Clínica de Digestivo, Servicio de Farmacología Clínica, Instituto de Investigación Biomédica de Málaga-IBIMA, Hospital Universitario Virgen de la Victoria, Universidad de Málaga, 29010 Málaga, Spain; 16 Liver Disease Laboratory and Liver Metabolism Laboratory, CIC bioGUNE, CIBERehd, Bizkaia Science and Technology Park, 48160 Derio, Bizkaia Spain; 17 The Liver Unit, Newcastle-upon-Tyne Hospitals NHS Foundation Trust, NE7 DN Newcastle upon Tyne, UK; 18 Newcastle University Translational and Clinical Research Institute, The Medical School, Newcastle University, NE7 DN Newcastle upon Tyne, UK; 19 Division of Gastroenterology, Hepatology, and Endoscopy, Department of Medicine, Brigham and Women´s Hospital, Harvard Medical School, Boston, and Harvard Digestive Diseases Center, 02115 Boston, MA USA; 20 Liver Vascular Biology Research Group, IDIBAPS, 08036 Barcelona, Spain; 21 Hepatology, Department of Biomedical Research, University of Bern, cH-3008, Bern, Switzerland; 22 Department of Internal Medicine III, University Hospital RWTH Aachen, 52074 Aachen, Germany

**Keywords:** Biomarkers, Pathogenesis

## Abstract

Acetaminophen (APAP) hepatotoxicity induces endoplasmic reticulum (ER) stress which triggers the unfolded protein response (UPR) in hepatocytes. However, the mechanisms underlying ER stress remain poorly understood, thus reducing the options for exploring new pharmacological therapies for patients with hyperacute liver injury. Eight-to-twelve-week-old C57BL/6J *Xbp1*-floxed (*Xbp1*^*f/f*^) and hepatocyte*-*specific knockout Xbp1 mice (*Xbp1*^*∆hepa*^) were challenged with either high dose APAP [500 mg/kg] and sacrificed at early (1–2 h) and late (24 h) stages of hepatotoxicity. Histopathological examination of livers, immunofluorescence and immunohistochemistry, Western blot, real time (RT)-qPCR studies and transmission electron microscopy (TEM) were performed. Pharmacological inhibition of XBP1 using pre-treatment with STF-083010 [STF, 75 mg/kg] and autophagy induction with Rapamycin [RAPA, 8 mg/kg] or blockade with Chloroquine [CQ, 60 mg/kg] was also undertaken in vivo. Cytoplasmic expression of XBP1 coincided with severity of human and murine hyperacute liver injury. Transcriptional and translational activation of the UPR and sustained activation of JNK1/2 were major events in APAP hepatotoxicity, both in a human hepatocytic cell line and in a preclinical model. *Xbp1*^*∆hepa*^ livers showed decreased UPR and JNK1/2 activation but enhanced autophagy in response to high dose APAP. Additionally, blockade of XBP1 splicing by STF, mitigated APAP-induced liver injury and without non-specific off-target effects (e.g., CYP2E1 activity). Furthermore, enhanced autophagy might be responsible for modulating CYP2E1 activity in *Xbp1*^*∆hepa*^ animals. Genetic and pharmacological inhibition of *Xbp1* specifically in hepatocytes ameliorated APAP-induced liver injury by enhancing autophagy and decreasing CYP2E1 expression. These findings provide the basis for the therapeutic restoration of ER stress and/or induction of autophagy in patients with hyperacute liver injury.

## Introduction

Because of its extensive worldwide use and narrow therapeutic window, acetaminophen (APAP) hepatotoxicity is considered a significant public health concern. Despite half a century of research, the specific mechanisms of APAP hepatotoxicity are still not fully understood, and clinical treatment is limited.

The endoplasmic reticulum (ER) is a major intracellular organelle that performs multiple physiological functions including protein folding, post-translational modifications, biosynthesis of fatty acids and sterols, detoxification of xenobiotics, and storage of intracellular Ca^2+^ [1]. Upon exposure to potential stressors such as drugs, the ER initiates the unfolded protein response (UPR) to restore homeostasis [2].

In mammalian cells, the UPR consists of three primary pathways that sense the accumulation of unfolded or misfolded proteins. These sensor protein-transcription factor pairs are: (i) Protein kinase RNA-like endoplasmic reticulum kinase (PERK) and eukaryotic Initiation Factor 2 alpha (eIF2α); (ii) the activating transcription factor 6 (ATF6); and (iii) Inositol requiring kinase 1α (IRE1α) and X-box binding protein 1 (XBP1) [3]. Upon activation of IRE1α, splicing of the *Xbp1* mRNA occurs, thus enabling the translation of a spliced XBP1 protein (sXBP1). sXBP1 is a transcription factor that induces genes involved in chaperoning proteins through the ER and degrading proteins that cannot be properly folded in the ER [4]. The role of ER stress in APAP-induced hepatocellular injury is supported by a considerable amount of data. APAP hepatotoxicity results in increased expression of phosphorylated eIF2a and CCAAT-enhancer-binding protein homologous protein (CHOP) [5], whilst sublethal doses of APAP activate the transcription factor ATF6 [6]. More importantly, the IRE1α-XBP1 arm of the UPR response has been shown to play a critical role in APAP-induced liver injury via the regulation of cytochrome P450 activity [1].

In the present study we sought to investigate the molecular mechanisms associated with high dose, APAP-induced hepatic ER stress in vivo and in vitro. Our study reveals a novel mechanism of protection against APAP hepatotoxicity by which decreased sXBP-1 induces autophagy.

## Materials and methods

### Human samples

Livers from patients undergoing urgent liver transplantation for ALF resulting from APAP overdose were evaluated for XBP1 expression in liver paraffin sections. The diagnosis of ALF was established in Newcastle Hospitals NHS Foundation Trust (Newcastle, UK). The samples were obtained by the Newcastle Biomedicine Biobank (12/NE/0395), and approved by Newcastle Research Ethics Committee North East Newcastle and North Tyneside. Patients´ clinicopathological characteristics are shown in Table 1. All patients gave informed consent for all clinical investigations, according to the principles embodied in the Declaration of Helsinki. Samples are explants from patients undergoing urgent liver transplantation for ALF, with a timing between paracetamol overdose and explant of within 12 h.

**Table 1:** Clinicopathological characteristics of the patients used in this study.

Patient	Age (yrs)	ALT (U/L)	ALP (U/L)	TBIL (µmol/L)	Inflammation/Infiltration	Steatosis
#1	22	1666	149	53		x
#2	44	3156	268	61	x	
#3	28	912	78	89		x
#4	39	1774	135	306	x	
#5	15	1369	86	71	x	
#6	44	4328	83	68	x	
#7	32	1151	171	224		
#8	56	685	76	178		
#9	47	2512	246	197	x	x
#10	38	4810	140	70	x	
#11	51	10048	120	123		
#12	38	2260	166	253		x
#13	19	2100	151	137		
${\over{{\rm{X}}}}$ ± SD	36.0 ± 12.6	2829.0 ± 2500.5	144.0 ± 60.4	141.0 ± 83.5		

*yrs* years, *ALT* alanine aminotransferase, *ALP* alkaline phosphatase, *TBIL* total bilirubin.

### Experimental models

All animals were randomly allocated to the experimental groups. Eight-to-twelve-week-old C57/BL6J mice purchased from ENVIGO (Valencia, Spain) were fasted overnight and challenged with an intraperitoneal (i.p.) injection of APAP [500 mg/kg]. Mice harboring a conditional floxed allele of *Xbp1* (*Xbp1*^*f/f*^), a gift from Richard S. Blumberg (Harvard Medical School) were crossed with Alb-Cre mice (Charles River España, Cerdanyola del Vallés, Barcelona) to obtain a liver specific knockout of *Xbp1* (*Xbp1*^*∆hepa*^). Overnight-fasted 8–12 week-old male *Xbp1*^*f/f*^ and *Xbp1*^*∆hepa*^ mice were challenged with an i.p. injection of APAP [500 mg/kg] or vehicle. Since it is a high dose and APAP is insoluble in water, APAP was first dissolved in DMSO then diluted to 50 mg/ml with PBS (final concentration of DMSO < 1%). Control mice received an equivalent i.p. volume of PBS with DMSO. For experiments using STF-083010 (STF, Eurodiagnóstico, Madrid, Spain), mice were injected i.p [75 mg/kg] or an equal volume of PBS with 10% DMSO (i.p) [7]. For autophagy interventional experiments, [8 mg/kg] rapamycin (RAPA, Palex Medical, Barcelona, Spain) or [60 mg/kg] chloroquine (CQ, Palex Medical) were i.p. injected 1 h prior to APAP challenged as previously reported [8].

Upon sacrifice, serum was collected from the inferior vena cava, and serum alanine aminotransferase (ALT), aspartate aminotransferase (AST) and lactate dehydrogenase (LDH) were measured in the Central Laboratory Facility at the Gregorio Marañón Research Institute, Madrid (IiSGM) using automated analyzers. Animals were bred and maintained in the Animal Facility of the Faculty of Medicine at UCM, Madrid, under pathogen-free conditions in a temperature and humidity-controlled room with 12 h light/dark cycles and allowed food and water ad libitum. Animal work was approved by the Conserjería de Medio Ambiente, Administración Local y Ordenación del Territorio (PROEX-218/17 and 125.1/20).

### Other methodology

For immunoblot analysis membranes were incubated overnight with the primary antibodies shown in Supplementary Table 1. GAPDH was used as a loading control. For RT-PCRs, human and mouse primers were purchased from Merck (sequences are shown in Supplementary Tables 2 and 3, respectively). Enzymatic colorimetric test was performed using the Triglycerides kit (RAL, Barcelona, Spain) for quantitative determination of triglycerides in the liver.

### Statistical analysis

Statistical analysis was performed using the software Prism v8.0 (GraphPad Software). HepaRG cells and C57BL/6J mice were compared using one-way analysis of variance (ANOVA) and statistical significance was assessed by two-way ANOVA adjusted for Tukey’s multiple comparisons. Data are expressed as means ± standard error of the mean (SEM). A *p* value equal or less than 0.05 was considered to be statistically significant.

## Results

### Increased XBP1 expression in human and murine APAP hepatoxicity

To investigate whether XBP1 could play a role in the UPR associated with APAP overdose, XBP1 expression was investigated in liver tissue sections obtained from patients undergoing emergency liver transplantation for APAP-induced ALF. The clinicopathological characteristics of these patients are summarized in Table 1.

**Fig. 1:**
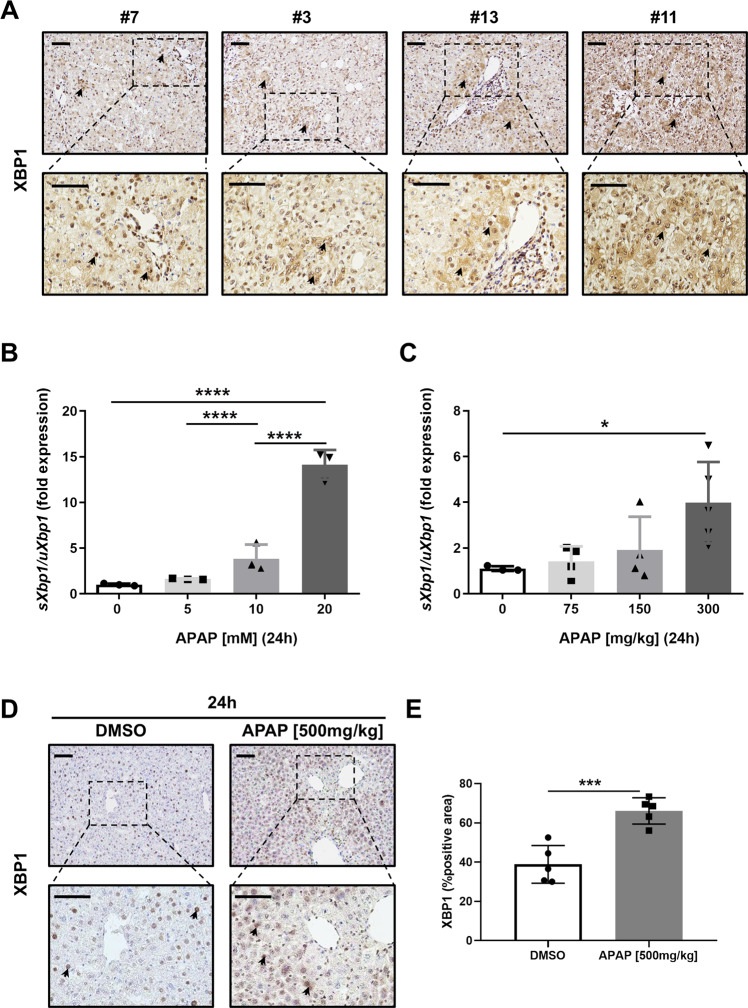
The expression of XBP1 increases during APAP overdose both in mouse and human. **A** Representative XBP1 staining performed on paraffin liver sections of patients with acute liver failure (ALF) due to APAP overdose. Scale bars, 100 μm (top) and 50 μm (bottom) (*N* = 13). **B** mRNA levels of sXBP1/uXBP1 in HepaRG cells, were determined by RT-qPCR, 24 h after APAP challenge. **C** mRNA level of sXBP1/uXBP1 in C57BL/6J wild-type (WT) mice was determined by RT-qPCR, 24 h after APAP injection. **D** Representative XBP1 staining was performed on paraffin liver sections of C57BL/6 wild-type (WT) mice, 24 h post-APAP [500 mg/kg]. Scale bars, 100 μm and 50 μm. **E** Quantification of XBP1-positive area was performed. Data are expressed as mean ± SEM and graphed, separately (*p < 0.05- ****p < 0.0001; *N* = 3–5 animals per experimental group).

Given that the antibody detects both the unspliced and the spliced forms of XBP1, most hepatocyte nuclei expressed XBP1. However, cytoplasmic expression of XBP1 became more evident as hepatocyte architecture was altered (Fig. 1A). These samples showed increased levels of serum markers of liver disease (Table 1). We first investigated the activation of the UPR response after exposure to APAP in a mycoplasma-free human hepatic cell line, HepaRG, and in a preclinical in vivo model. Interestingly, we found that splicing of *Xbp1* occurred under high concentrations of APAP in vitro (Fig. 1B) and in a murine model after high dose APAP (Fig. 1C).

Additionally, we evaluated the expression of XBP1 by IHC staining in liver tissues from mice treated with either vehicle or high-dose APAP [500 mg/kg] for 24 h (Fig. 1D). Positive staining for XBP1 was evident in hepatocyte nuclei in control-vehicle treated mice. However, 24 h after high-dose APAP triggered significantly increased XBP1 expression in the liver parenchyma, and specifically in hepatocyte cytosols (Fig. 1D, E), suggesting that transcriptional activation of XBP1 is an event directly related with the UPR in human and murine APAP-induced liver injury.

### *Xbp1*^*∆hepa*^ mice show decreased hepatic UPR and JNK1/2 activation in response to APAP

**Fig. 2:**
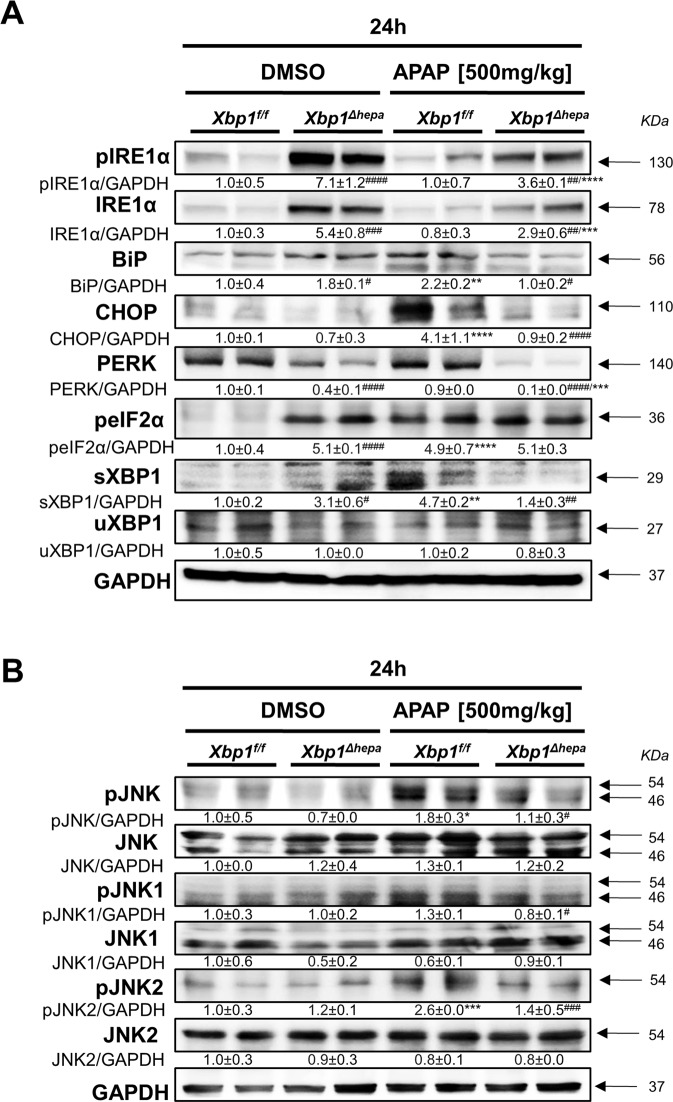
Consequences of ablation of XBP1 in hepatocytes in the UPR and JNK activation, 24 h after high dose APAP. **A** Protein levels of pIRE1α, IRE1α, BiP, CHOP, peIF2α, PERK, sXBP1, and uXBP1, were determined. **B** Protein levels of pJNK, JNK, pJNK1, JNK1, pJNK2, JNK2 were determined. GAPDH was used as a loading control. Relative protein levels were quantified using ImageJ software. Data are expressed as mean ± SEM (<0.05-****p < 0.0001; ^#^*p* < 0.05^##^*p* < 0.0001;*intragroup,^#^intergroup).

Since our results evidenced splicing of XBP1 in APAP intoxication for the first time in human explants and hepatocyte cell line, we generated mice with specific deletion of *Xbp1* in hepatocytes (*Xbp1*^*∆hepa*^). Littermate *Xbp1*^*f/f*^ mice were used as controls. Interestingly, livers from untreated *Xbp1*^*∆hepa*^ mice displayed significant hyperactivation of IRE1α and loss of XBP1 protein (Supplementary Fig. 1A), whilst loss of *Xbp1* in hepatocytes caused hyperactivation of IRE1α, as previously shown [1]. Therefore, we next sought to investigate the UPR after high-dose APAP in *Xbp1*^*∆hepa*^ mice. Whilst APAP did not greatly increase IRE1α phosphorylation in *Xbp1*^*f/f*^, phospho-IRE1α protein levels were overexpressed in mice with hepatocyte-specific deletion of *Xbp1* (*Xbp1*^*∆hepa*^) (Fig. 2A). In turn, 24 h post-APAP, an increase in CHOP and BiP/GRP78 protein levels in *Xbp1*^*f/f*^ compared with *Xbp1*^*∆hepa*^ livers was observed (Fig. 2A). Additionally, PERK protein levels were decreased in both untreated, and to a greater extent, in APAP-treated *Xbp1*^*∆hepa*^ mice, compared with *Xbp1*^*f/f*^ livers. Interestingly, peIF2 was absent in untreated *Xbp1*^*f/f*^ but significantly overexpressed in *Xbp1*^*∆hepa*^ livers. APAP induced also the expression of peIF2 in both *Xbp1*^*f/f*^ *and Xbp1*^*∆hepa*^ mice (Fig. 2A). As expected, the levels of uXBP1 were not altered by high-dose APAP; however, sXBP1 protein was overexpressed in *Xbp1*^*f/f*^ compared with *Xbp1*^*∆hepa*^ livers (Fig. 2A).

JNK is activated by APAP and plays a crucial role in hepatotoxicity [9–11]. To determine the effect of XBP1 ablation on JNK activation, we examined the JNK signaling pathway in liver tissues by immunoblotting, 24 h after high-dose APAP. Increased levels of pJNK were found in *Xbp1*^*f/f*^ mice, whereas lack of JNK induction was characteristic of *Xbp1*^*∆hepa*^ livers at this time point (Fig. 2B). Moreover, JNK1 and JNK2 phosphorylation was slightly decreased in *Xbp1*^*∆hepa*^ compared with *Xbp1*^*f/f*^ (Fig. 2B).

### Ablation of *Xbp1* in hepatocytes reduces hepatotoxicity both at early and late stages of high-dose APAP

**Fig. 3:**
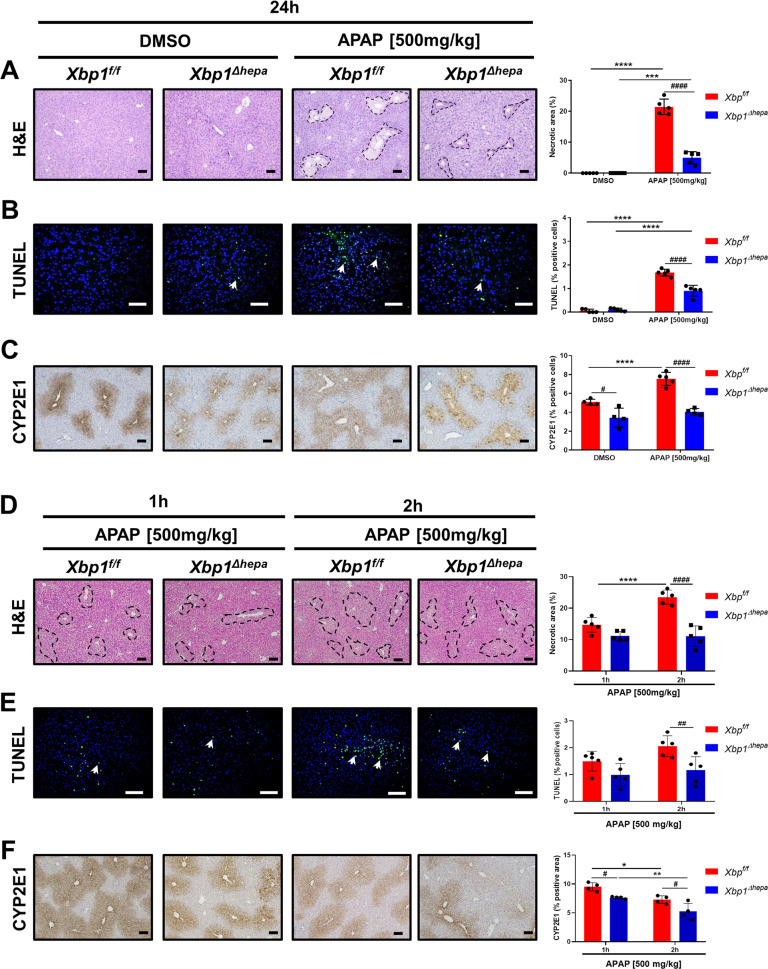
Ablation of Xbp1 in hepatocytes reduces hepatotoxicity both at early and late stages of high dose APAP. *Xbp1*^*f/f*^ and *Xbp1*^*∆hepa*^ mice were challenged with APAP [500 mg/kg] at late (24 h) and early (1–2 h) stages of hyperacute liver injury. **A** Representative H&E staining was performed in paraffin liver sections of *Xbp*^*f/f*^ and *Xbp1*^*∆hepa*^ animals, 24 h post-APAP, Necrotic foci were quantified. Scale bars, 100 μm. **B** Representative TUNEL staining performed on frozen liver sections. TUNEL-positive cells per view field were quantified. Arrows denote positive cells. Scale bars, 100 μm. **C** Representative IHC staining for CYP2E1 for the same samples and quantification of CYP2E1 area per view field. Scale bars, 100 μm. **D** Representative H&E staining was performed in paraffin liver sections, 1 and 2 h after APAP injury and necrotic foci were quantified. Scale bars, 100 μm. **E** Representative TUNEL staining performed on frozen liver sections of the same animals. TUNEL-positive cells per view field were quantified. Arrows denote positive cells. Scale bars, 100 μm. **F** Representative IHC staining for CYP2E1 for the same samples. Quantification of positive area per view field using Image J was performed. Scale bars, 100 μm. Data are expressed as mean ± SEM (*p < 0.05-****p < 0.0001; ^#^*p* < 0.05-^###^*p* < 0.001; *intragroup,^#^ intergroup; *N* = 5–6 per experimental group).

Concomitant to our results with APAP-derived hyperacute liver injury, marked splicing of XBP1 has been previously reported in mice, 24 h after high dose APAP [5]. Thus, we sought to thoroughly investigate the relevance of hepatocytic XBP1 in the pathophysiology of APAP-hepatotoxicity. Whilst APAP induced significant hyperacute liver injury in *Xbp1*^*f/f*^ animals, *Xbp1*^*∆hepa*^ mice displayed only a modest induction of serum ALT, AST and LDH levels, 24 h post-APAP (Supplementary Fig. 1B–D). Macroscopic and histological analyses of the liver revealed markedly reduced liver injury in *Xbp1*^*∆hepa*^ mice compared with *Xbp1*^*f/f*^ animals which exhibited severe centrilobular necrosis (Fig. 3A and Supplementary Fig. 1E), and significantly higher TUNEL-positive cells and cleavage of caspase-3 (CC3) (Fig. 3B and Supplementary Fig. 1F). APAP-induced hyperacute liver injury is initiated by N-acetyl-p-benzoquinone imine (NAPQI), generated by several cytochrome P450 (CYP) isoenzymes, primarily, CYP2E1 [12]. CYP2E1 protein levels visibly increased after high-dose APAP in *Xbp1*^*f/f*^ mice, but with clearly reduced expression under basal conditions (DMSO, vehicle), and 24 h post-APAP in *Xbp1*^*∆hepa*^ compared with *Xbp*^*f/f*^ mice (Fig. 3C and Supplementary Fig. 1F).

Next, experiments for early time points of APAP hepatotoxicity were performed to examine the contribution of CYP2E1 at this stage. We thus challenged *Xbp1*^*f/f*^ and *Xbp1*^*∆hepa*^ mice with high-dose APAP for 1 h and 2 h, and evaluated necrotic foci, cell death and CYP2E1 expression. Interestingly, 1 h post high-dose APAP challenge, triggered a tendency towards decreased hepatic injury and CYP2E1 expression, which was significant at the 2 h time-point in *Xbp1*^*∆hepa*^ compared with *Xbp1*^*f/f*^ mice (Fig. 3D, E and Supplementary Fig. 2A–C).

CYP2E1 metabolism leads to the generation of reactive oxygen species (ROS) including lipid peroxidation, which can be measured as 4-HNE adducts using immunohistochemistry (Supplementary Fig. 3A), and triglyceride accumulation, a well-established consequence of severe ER stress [13], both in liver tissue and in serum (Supplementary Fig. 3B–D). All these parameters were significantly reduced in *Xbp1*^*∆hepa*^ compared with *Xbp1*^*f/f*^ mice, 24 h after high-dose APAP. In addition, APAP treatment caused a significant upregulation of heme oxygenase-1 (*Ho-1*) in *Xbp1*^*f/f*^ compared with unaltered levels of this gene in *Xbp1*^*∆hepa*^ mice (Supplementary Fig. 3E).

Increased ROS disrupts hepatic tight junctions (TJs) [14, 15]. Both *Xbp1*^*f/f*^ and *Xbp1*^*∆hepa*^ mice treated with DMSO (vehicle) maintained normal cell polarity and adhesion as shown by ZO-1 western blots (Supplementary Fig. 1F). However, APAP caused a dramatic decrease of ZO-1 expression in *Xbp1*^*f/f*^ and to a lesser extent in *Xbp1*^*∆hepa*^ mice (Supplementary Fig. 1F). Additionally, the number of CD45 and CD11b was significantly reduced in *Xbp1*^*∆hepa*^ mice, and a tendency towards reduced number of F4/80 cells was found (Supplementary Fig. 4A–D). Concomitantly, reduced expression of mRNA transcripts for *Tnfα* in *Xbp1*^*∆hepa*^ indicated significantly decreased inflammation compared with *Xbp1*^*f/f*^ livers (Supplementary Fig. 4E), as well as markers of inflammasome activation (eg *IL-1β* and *Nrlp3*) were significantly lower in *Xbp1*^*∆hepa*^ (Supplementary Fig. 4E).

### Loss of *Xbp1* in hepatocytes promotes autophagy after APAP treatment

TEM analysis indicated the accumulation of abundant autophagosomes in livers of *Xbp1*^*∆hepa*^ mice following APAP treatment, whilst the hepatic parenchyma of *Xbp1*^*f/f*^ animals was predominantly necrotic (Supplementary Fig. 5).

**Fig. 4:**
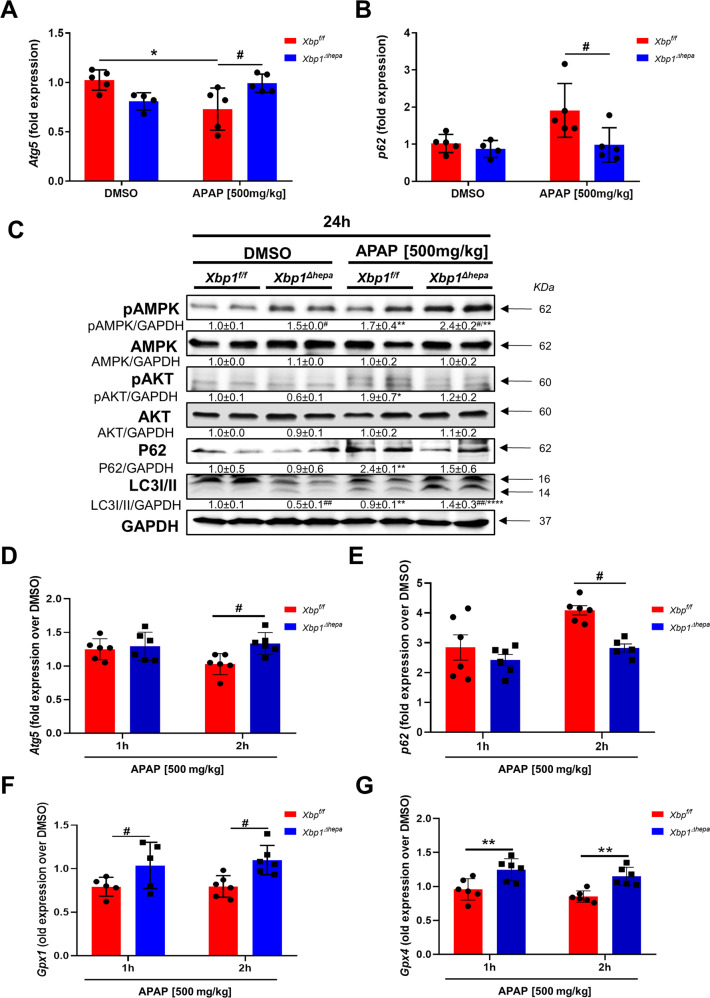
Loss of Xbp1 in hepatocytes promotes autophagy after APAP treatment. *Xbp1*^*f/f*^ and *Xbp1*^*∆hepa*^ mice were challenged with APAP [500 mg/kg] at late (24 h) and early (1–2 h) stages of hyperacute liver injury. **A**, **B** mRNA levels of *Atg5* (**A**) and *p62* (**B**) were determined by RT-qPCR, 24 h post-APAP. **C** Protein levels of pAMPK, AMPK, pAKT, AKT, P62, and LC3I/II were analyzed by western blot in liver extracts of the same mice. GAPDH was used as a loading control. Relative protein levels were quantified using ImageJ software. **D**–**G** mRNA levels of *Atg5* (**D**), *p62* (**E**), *Gpx1* (**F**), and *Gpx4* (**G**) were determined by RT-qPCR. Data are expressed as mean ± SEM (*N* = 4–5 per experimental group; intragroup,^#^ intergroup; *p* < 0.05-****p < 0.0001;^#^p < 0.05-^###^p < 0.0001).

ER stress activation is frequently accompanied by Ca^2+^ release and autophagy via the stimulation of AMPK, which leads to inhibition of the AKT/mTORC1 pathway, upregulation of the autophagy marker *Atg5* and degradation of *P62*. This was characteristic of *Xbp1*^*∆hepa*^ compared with *Xbp1*^*f/f*^ livers, 24 h after high-dose APAP (Fig. 4A, B). Immunoblotting confirmed increased protein levels of pAMPK in livers of *Xbp1*^*∆hepa*^ mice, thus attenuating pAKT, and promoting the degradation of P62 and the conversion of microtubule-associated protein light chain 3 (LC3)-I to LC3-II associated with autophagosome formation and autophagy (Fig. 4C).

Since decreased liver injury (eg necrosis) and impaired CYP2E1 activity was also observed in *Xbp1*^*∆hepa*^ animals, 1–2 h following high-dose APAP, we examined whether autophagy in these animals was also enhanced at early stages of hyperacute liver injury. Significantly increased *Atg5* mRNA transcripts and decreased *P62* mRNA expression was found at 2 h—but not at 1 h—after high-dose APAP in *Xbp1*^*∆hepa*^ livers (Fig. 4D, E**)**. Moreover, we explored changes in enzymes of the glutathione (GSH) system, including glutathione peroxidase-1 (GPX1) and -4 (GPX4) which were restored at early stages (1–2 h) of APAP hepatoxicity in *Xbp1*^*∆hepa*^ but depleted in *Xbp1*^*f/f*^ mice (Fig. 4F, G).

### Pretreatment with STF-083010 mitigated liver injury induced by high dose APAP

**Fig. 5:**
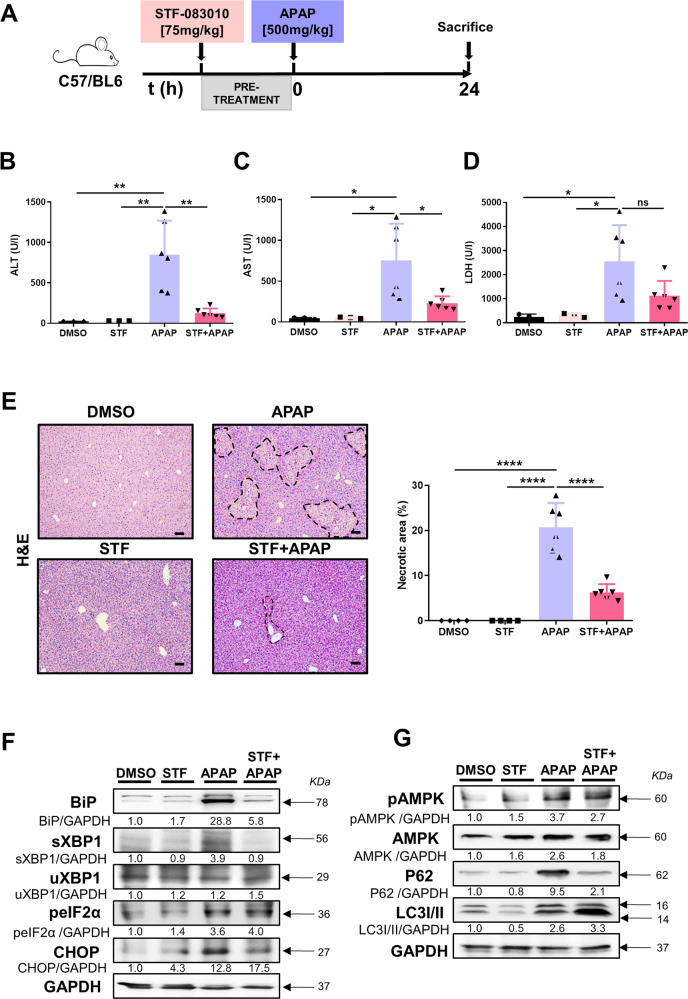
Pretreatment with STF-083010 (STF) attenuates APAP-induced hepatic injury and the UPR, and promotes autophagy after challenge with APAP. **A** C57BL/6J mice were injected i.p. with STF [75 mg/kg] or an equal volume of PBS with 10% DMSO, 1 h before APAP [500 mg/kg] and sacrificed 24 h later. A control group of STF-injected mice, was included. **B**–**D** Markers of liver damage were measured and represented. Serum ALT (**B**), AST (**C**) and LDH (**D**) levels represented as U/L. **E** Representative H&E staining was performed in paraffin liver sections and necrotic foci were quantified. Scale bars, 100 μm. **F** Protein levels of BiP, CHOP, peIF2α, sXBP1, and uXBP1 were determined. **G** Protein levels of pAMPK, AMPK, P62, and LC3I/II were determined. Data are expressed as mean ± SEM (*N* = 4–5 per experimental group; **p < 0.01-****p < 0.0001;* intragroup.

STF-083010 (STF), an inhibitor of the endonuclease activity of IRE1α, blocks the splicing of XBP1 in vivo and thus prevents the initiation of the UPR [16]. Therefore, our next experimental approach was to pharmacologically mimic the protection from APAP-mediated liver injury observed in *Xbp1*^*∆hepa*^ mice. C57BL/6J mice were pretreated with STF-083010 and sacrificed 24 h after high-dose APAP. A significant amelioration of serum levels of ALT and AST (Fig. 5A–C), and a tendency towards decreased LDH levels (Fig. 5D) was observed in STF + APAP-treated mice. Blinded histopathological evaluation of liver tissue demonstrated that STF manifestly mitigated APAP-induced hepatic necrosis and inflammation (Fig. 5E). Moreover, STF markedly attenuated APAP-induced overexpression of BiP, CHOP, and sXBP1 but had no effect on peIF2α protein levels (Fig. 5F). Interestingly, STF promoted a slight activation of AMPK, and the degradation of P62 and the conversion of LC3-I to LC3-II (Fig. 5G).

### STF-083010 post-treatment does not modify APAP-induced CYP2E1 activity

**Fig. 6:**
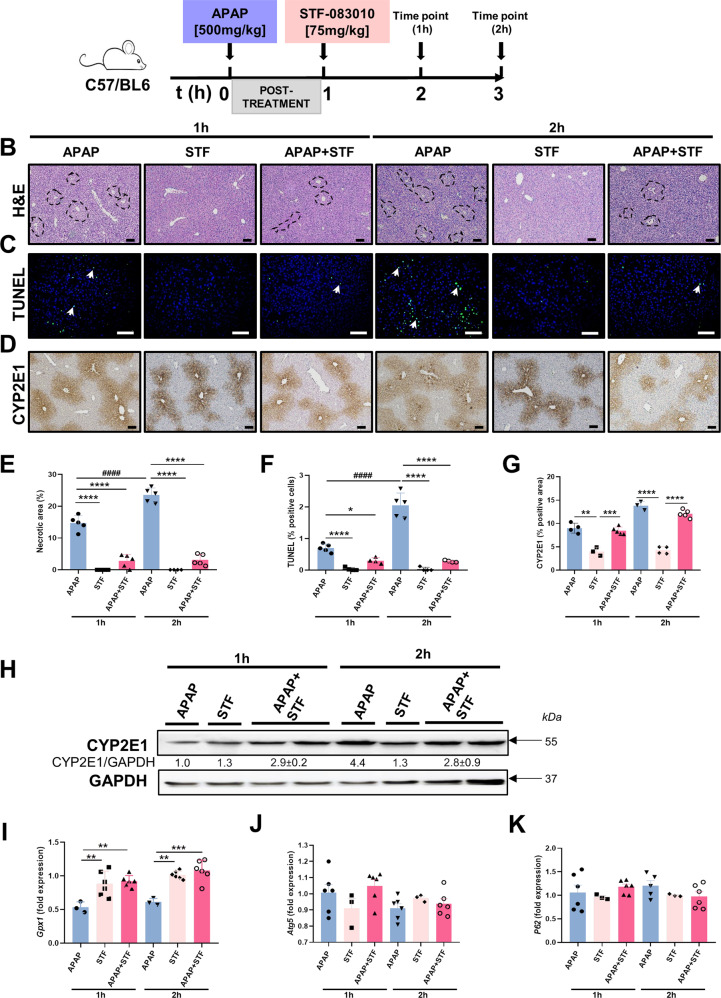
Post-treatment with STF does not modify CYP2E1 expression during APAP hepatoxicity. **A** C57BL/6J mice were injected APAP [500 mg/kg], and 1 h later STF [75 mg/kg] or an equal volume of PBS with 10% DMSO. Mice were sacrificed 1 and 2 h later. A control group was included with only STF-injected mice. **B** Representative H&E staining was performed in liver paraffin sections. Scale bars, 100 μm. **C** Representative TUNEL staining was performed on frozen liver sections Arrows denote positive cells. Scale bars, 100 μm. **D** Representative IHC staining for CYP2E1. Scale bars, 100 μm. **E** Necrotic foci were quantified and graphed. **F** TUNEL-positive cells were quantified and graphed. **G** Positive area for CYP2E1 staining was calculated using ImageJ and graphed. **H** Protein levels of CYP2E1 were determined using western blot. GAPDH was used as a loading control. Relative protein levels were quantified using ImageJ software. **I**–**K** mRNA expression was analyzed for *Gpx1* (**I**), *Atg5* (**J**), *p62* (**K**), and were evaluated using qRT-PCR. Data are expressed as mean ± SEM (*p < 0.05-****p < 0.0001;* intragroup; *N* = 3–6 per experimental group).

In order to rule out possible off-target effects of STF, we injected STF at 1–2 h after high-dose APAP (Fig. 6A). APAP significantly increased the percentage of necrotic area, TUNEL-positive cells and CYP2E1 activity at early stages (1–2 h), whilst STF treatment had no effect on these parameters. In contrast, high-dose APAP and post-treatment with STF (APAP + STF) at 1 and 2 h, significantly decreased the percentage of necrotic areas and TUNEL-positive cells, but had no impact on CYP2E1 protein expression (Fig. 6B–G). These results were validated by immunoblot, where STF alone or APAP + STF had a greater impact on CYP2E1 expression after 2 h (Fig. 6H). Finally, while APAP + STF was capable of restoring *Gpx1* depletion, STF post-treatment did not change the mRNA expression of *Atg5* or *P62* (Fig. 6I–K).

### Autophagy modulation and CYP2E1 expression in *Xbp1*^*Δhepa*^ animals

**Fig. 7:**
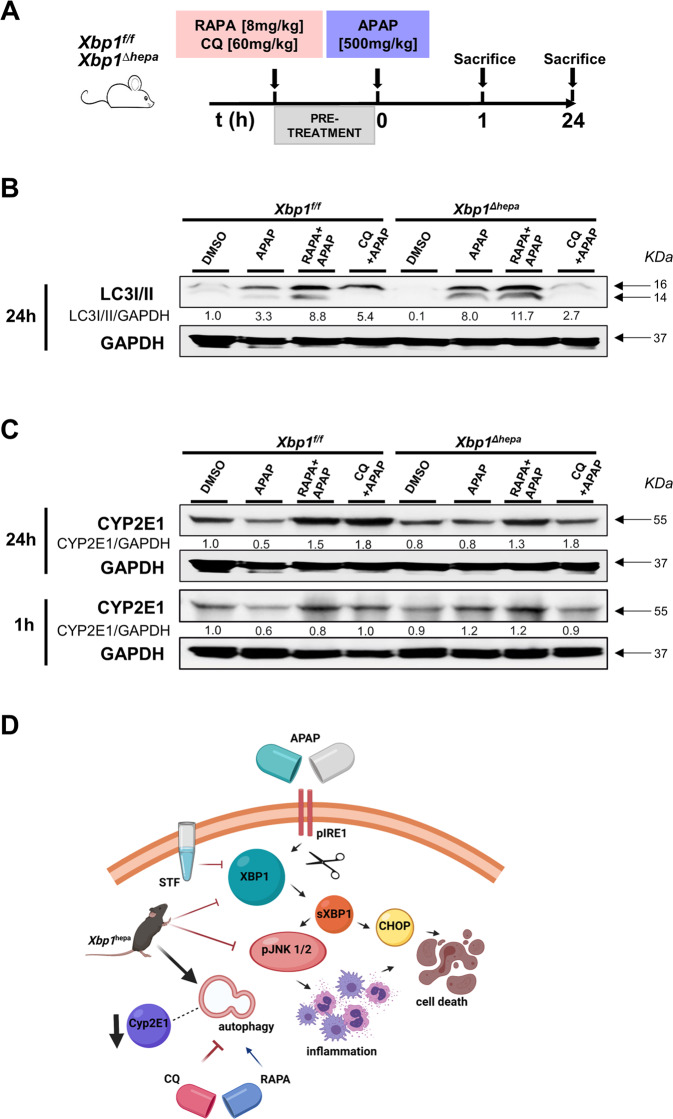
Autophagy intervention using rapamycin (RAPA) or chloroquine (CQ) during early and late APAP-derived hyperacute liver injury. **A** *Xbp1*^*f/f*^ and *Xbp1*^*∆hepa*^ mice were intraperitoneally injected with either rapamycin (RAPA) [8 mg/kg] or chloroquine **(**CQ) [60 mg/kg], 1 h prior to APAP [500 mg/kg] challenge, and sacrificed at early (1 h) or late times (24 h) after APAP toxicity. **B** Protein levels of LC3I/II were determined in *Xbp1*^*f/f*^ and *Xbp1*^*∆hepa*^ mice, pretreated with RAPA or CQ and sacrificed 24 h APAP challenge. **C** Protein levels of CYP2E1 were determined in *Xbp1*^*f/f*^ and *Xbp1*^*∆hepa*^ mice, pretreated with RAPA or CQ and sacrificed 24 h (top panel) –same membrane used as in (**B**) and 1 h (bottom panel) post-APAP. GAPDH was used as a loading control. Relative protein levels were quantified using ImageJ software. **D** Schematic representation of the effects of genetic deletion of XBP1 in hepatocytes and pharmacological intervention, during APAP hepatotoxicity.

Given that hepatocyte-specific deletion or pharmacological blocking of XBP1 splicing might trigger autophagy activation, we further evaluated the consequences of inducing or blocking autophagy with drugs in our experimental setting. Because suppression of mTOR is a central molecular signaling pathway leading to autophagy induction [17], we used rapamycin (RAPA). In contrast, chloroquine (CQ), a well-known lysosomal inhibitor was utilized as an autophagy blocking agent. Both RAPA and CQ were administered i.p. 1 h prior to high-dose APAP to both *Xbp1*^*f/f*^ and *Xbp1*^*Δhepa*^ mice and sacrifice took place at early (1 h) and late stages (24 h) of hyperacute liver injury (Fig. 7A). APAP challenge induced LC3-I and mildly LC3-II in *Xbp1*^*f/f*^ livers, which was accentuated by RAPA, which strongly caused overexpression of LC3-I/II (Fig. 7B). In sharp contrast, APAP clearly triggered overexpression of LC3-I/II in *Xbp1*^*Δhepa*^ livers with no effect observed in RAPA-pretreated *Xbp1*^*Δhepa*^ mice. Interestingly CQ pre-treatment ameliorated autophagy both in *Xbp1*^*f/f*^ and more clearly in *Xbp1*^*f/f*^ animals (Fig. 7B).

We previously showed that *Xbp1*^*Δhepa*^ mice have decreased protein levels of CYP2E1 in basal conditions (Fig. 3C and Supplementary Fig. 1F). Thus, we speculated that autophagy could prevent from CYP2E1-mediated hepatotoxicity to *Xbp1*^*Δhepa*^ mice. APAP challenged decreased CYP2E1 levels in *Xbp1*^*f/f*^ mice, a phenomenon reverted by pretreatment with RAPA or CQ. Expectedly, *Xbp1*^*Δhepa*^ livers displayed decreased basal CYP2E1 levels, which were barely induced by RAPA nor by CQ both at early (1 h) and late (24 h) stages of APAP hepatotoxicity (Fig. 7C), indicating that autophagy might be responsible for suppressed CYP2E1 activity in hepatocyte-specific *Xbp1* knockout mice.

## Discussion

Although APAP hepatotoxicity remains the leading cause of acute liver failure (ALF), temporal and quantitative effects of direct APAP-mediated liver injury on the induction of endoplasmic reticulum (ER) stress remain poorly understood. Activated IRE1 splices the mRNA of XBP1 in a non-canonical fashion, yielding the potent transcription factor sXBP1 [18, 19].

Interestingly, we found that whilst most hepatocyte nuclei expressed XBP1, cytoplasmic expression of XBP1 became more evident as hepatocyte architecture was altered and serum transaminases increased in patients with APAP overdose. Mechanisms of APAP hepatotoxicity have been reported to be similar in both humans and mice [14]. Indeed, mRNA expression of spliced/unspliced (sXBP1/uXBP1) in human hepatic HepaRG cells was consistent with our findings in murine APAP hepatotoxicity.

Our data also showed that hepatic deletion of *Xbp1* leads to marked hyperactivation of IRE1α as previously reported [1, 20]. Moreover, we found that reduced CHOP, BiP/GRP78 and JNK activation were involved in protection against APAP-induced hepatotoxicity. Additionally, protection from APAP through activation of regulated IRE1-dependent decay (RIDD) might play a further role in the protection against APAP, since enhanced RIDD leads to pro-death signaling by reducing the levels of select microRNAs that normally repress pro-apoptotic targets [21]. Furthermore, decreased sXBP1 was linked to the suppression of cytochrome P450 activity [3].

While our data obtained in wildtype mice mirrors previous reports indicating that CYP2E1 activity increases at early time points (1–4 h), and then increases only at 24 and 96 h after APAP in rodents [22, 23], *Xbp1*^*∆hepa*^ livers were characterized by decreased CYP2E1 both at early and late phases of APAP hepatoxicity. This was linked to restoration of the GSH enzymes, preventing the severe oxidative stress occurring in normal mice. These data also correlated with the cytoprotective effect—reduced number of necrotic areas and decreased cell death—of *Xbp1*-deficient mice, at early and late stages after high dose APAP.

In recent years, accumulating evidence has suggested that autophagy is activated against APAP-induced liver injury [24]. In our mice, TEM showed hepatocytes had normal ER ultrastructure in *Xbp1*^*∆hepa*^ mice, but abundant autophagosome formation and less mitochondrial damage upon APAP toxicity. Additionally, hepatocytic *Xbp1* deficiency significantly promoted the phosphorylation of AMPK, augmented the effect of high dose APAP on the induction of LC3-II. Moreover, while *Atg5* levels were increased, the levels of P62 were decreased both at early and late stages of APAP hepatoxicity, indicating that APAP-induced autophagy in the liver was a consequence of *Xbp1* deletion.

STF-083010 (STF), a specific inhibitor of IRE1α RNase, inhibits both endogenous and chemically-induced *Xbp1* splicing without affecting IRE1α phosphorylation. Pharmacological inhibition of STF not only markedly reduced the levels of serum transaminases, but also mitigated liver injury and prevented oxidative burst induced by high-dose APAP in mice. This is in agreement with previous reports demonstrating anti-oxidant and anti-inflammatory effects of STF [21, 25]. Moreover, our study demonstrated that STF has a protective effect against APAP hepatotoxicity, and promoted the activation of autophagy, without affecting CYP2E1 activity. Our data are also in agreement with previous studies showing that pretreatment with STF facilitated hepatocyte autophagy in response to toxin stimulation [26].

We next evaluated whether induction or pharmacological blocking of autophagy using Rapamycin (RAPA) and Chloroquine (CQ), respectively, might affect CYP2E1 levels. Our results suggest that reduced CYP2E1-associated drug metabolism is associated with enhanced autophagy in the absence of hepatocytic XBP1. These data were similar at early and late stages of APAP hepatotoxicity.

In summary, this study provides new mechanistic information on the role of ER stress, UPR and autophagy in APAP-mediated hepatotoxicity (Fig. 7D). Altogether, our study confirms previous observations [27] suggesting that pharmacological modulation of autophagy, including decreasing expression of the transcription factor sXBP1, a key regulator of the UPR, may be a novel therapeutic approach for treating APAP-induced liver injury.

## Supplementary information

Suppl. Material

Suppl. Fig. 1

Suppl. Fig. 2

Suppl. Fig. 3

Suppl. Fig. 4

Suppl. Fig. 5

checklist

## Data Availability

Datasets used or analyzed in the current study are available from the corresponding author upon reasonable request.
