# Supplementary material for: Genetic and pharmacological inhibition of XBP1 protects against APAP hepatotoxicity through the activation of autophagy
Source: Cell Death Dis. 2022 Feb 10;13(2):143. doi: 10.1038/s41419-022-04580-8 (PMC8831621; doi:10.1038/s41419-022-04580-8)
Supplement: Supplementary file 1 — Suppl. Material [file 41419_2022_4580_MOESM1_ESM.docx]

**SUPPLEMENTARY MATERIAL**

**Genetic and pharmacological inhibition of XBP1 protects against APAP hepatotoxicity through the activation of autophagy**

Hui Ye, Chaobo Chen, Hanghang Wu, Kang Zheng, Beatriz Martín-Adrados, Esther Caparros, Rubén Francés, Leonard J. Nelson, Manuel Gómez del Moral, Iris Asensio, Javier Vaquero, Rafael Bañares, Matías A. Ávila, Raúl J. Andrade, M. Isabel Lucena, Maria Luz Martínez-Chantar, Helen L. Reeves, Steven Masson, Richard S. Blumberg, Jordi Gracia-Sancho, Yulia A. Nevzorova, Eduardo Martínez-Naves and Francisco Javier Cubero

**SUPPLEMENTARY METHODS**

**Immunoblot analysis**

Protein extracted from liver samples in NP40 buffer were loaded on handmade gels and transferred onto polyvinylidene difluoride (PVDF) or nitrocellulose membranes. Primary antibodies were detected with anti-mouse (Biorad, Madrid, Spain) or anti-rabbit (Werfen, Barcelona, Spain) IgG antibodies and signals were developed using Amersham ECL Prime (GE Healthcare, Madrid, Spain).

**Histological evaluation and immunofluorescence** **staining**

Paraffin-embedded hepatic tissue was sectioned and stained for H&E. Samples were examined by a pathologist blinded who analyzed the degree of liver injury. The percentage of necrotic area fraction in H&E staining was quantified on 10 to 20 low-power (magnification, x10) fields per slide, using NIH ImageJ® software (<http://rsbweb.nih.gov/>). The coverslips or 8 µM thick frozen sections of liver samples were also stained in 0.2% Oil Red O (ORO) and counterstained with hematoxylin.

Immunohistochemistry on paraffin sections was performed. Briefly, liver sections were deparaffinized with xylene and rehydrated with serially descending percentages of ethanol. The sections were then boiled in 10 mM sodium citrate acid buffer (pH=6) to enhance the availability of the antigen followed by incubation with 3% H_2_O_2_. Afterwards, the sections were subjected to 2.5% horse serum (Palex Medical) and incubated overnight with CYP2E1 and XBP1 (Abcam) antibodies at 4°C. The next day, slides were incubated with secondary antibody (Palex Medical) for 1h at RT in a humidifying box. The signal was developed with Diaminobenzidine (DAB, peroxidase substrate kit) (Palex Medical). The sections were counterstained using hematoxylin and mounted with Roti-histokit (Quimivita, Barcelona, Spain).

For the immunofluorescence staining, frozen cryosections were incubated with CD11b (BD Biosciences, Madrid, Spain), CD45 (BD Biosciences), F4/80 (Biorad) or ZO-1 (Fisher Scientific) overnight and incubated with fluorescence labeled secondary antibodies (AlexaFluor 488, Fisher Scientific). Slides were then mounted with DAPI (Palex Medical) and imaged using Axio Imager A1 microscope (Carl Zeiss AG, Jena, Germany) and AxioVision software. The cryosections from mouse liver were also incubated with *in situ* cell death detection kit (Roche, Madrid, Spain) overnight at 4 ºC.

**RNA isolation and quantitative real-time polymerase chain reaction (RT-PCR)**

Total cellular RNA was isolated with Trizol (Fisher Scientific). For reverse transcription, 1 µg of total RNA was transcribed using Applied Biosystems™ High-Capacity cDNA Reverse Transcription Kit (Fisher Scientific). Quantitative real-time PCR was carried out by a Real Time PCR machine employing Sybr Green PCR Master Mix (Fisher Scientific). The Ct values were extrapolated to a standard curve and data was normalized to the house-keeping gene expression (GAPDH).

**Transmission electron microscopy (TEM)**

Liver tissues were fixed with 4% formaldehyde and 2.5% glutaraldehyde in phosphate solution (pH 7.2-7.4) overnight, post-fixed with 1% osmium tetroxide in double-distilled water for 1h, dehydrated through graded acetone, and embedded in resin. Ultrathin sections were cut with a diamond knife on an ultramicrotome. The thin sections were stained and observed using a TEM Jeol 1010 (Jeol, Peabody, USA).

**SUPPLEMENTARY TABLES**

**Suppl. Table 1. Primary antibodies used.**

| **Antibody** | **Manufacturer** | **Catalog** |
| --- | --- | --- |
| p-AKT | Werfen, Barcelona, Spain | 9271S |
| AKT | Werfen | 9272S |
| p-AMPK | Werfen | 2535S |
| AMPK | Werfen | 2532S |
| BIP | Werfen | 3177S |
| CYP2E1 | Abcam, Cambridge, UK | ab28146 |
| CHOP | Werfen | 2895S |
| Cleaved caspase 3 | Werfen | 9661S |
| p-JNK | Werfen | 9251S |
| JNK | Werfen | 9252S |
| p-JNK1 | Bionova científica, Madrid, Spain | NB100-82009 |
| JNK1 | Werfen | 3708S |
| p-JNK2 | Bionova científica | NBP1-45787 |
| JNK2 | Werfen | 4572S |
| LC3I/II | Werfen | 12741T |
| P62 | Werfen | 39749S |
| PERK | Werfen | 3192 |
| p-eIF2α | Werfen | 3398 |
| p-IRE1α | Bionova científica | NB100-2323 |
| IRE1α | Werfen | 3294S |
| spliced XBP1 | Werfen | 12782S |
| XBP1 | Abcam | ab220783 |
| XBP1 | Abcam | ab37152 |
| ZO-1 | Fisher Scientific, Madrid, Spain | 61-7300 |
| GAPDH | Biorad | MCA4739 |

**Suppl. Table 2. Human primers used.**

| **Gene** | **Forward** | **Reverse** |
| --- | --- | --- |
| *sXbp1* | CGCTTGGGGATGGATGCCCTG | CCTGCACCTGCTGCGGACT |
| *uXbp1* | TGGCCGGGTCTGCTGAGTCCG | ATCCATGGGGAGATGTTCTGG |
| *Gapdh* | CAAGGTCATCCATGACAACTTTG | GTCCACCACCCTGTTGCTGTAG |

**Suppl. Table 3. Mouse primers used.**

| **Gene** | **Forward** | **Reverse** |
| --- | --- | --- |
| *Atg5* | ACAGCTTCTGGATGAAAGGC | TGGGACTGCAGAATGACAGA |
| *Gapdh* | TGTTGAAGTCACAGGAGACAACCT | AACCTGCCAAGTATGATGACATCA |
| *ho-1* | GAATCGAGCAGAACCAGCCT | GCCTTCTCTGGACACCTGAC |
| *Il-1β* | TGTGTG ACGTTCCCATT | CAGCACGAGGCTTTTTTGTTG |
| *Ire1α* | TGC TCA AGG ACA TGG CTA CCA TTA | CTGGAACTGTTGGTGCTGGA |
| *Nlrp3* | TGTGAGAAGCAGGTTCT CTC T | TGT AGC GAC TGT TGA GGT CCA |
| *p62* | AGA ATGTGGGGGAGAGTGTG | TCGTCTCCTCCTGAGCAGTT |
| *Tnfα* | CCTCTTCTCATTCCTGCTTGTGG | GAGAAGATGATCTGAGTGTGAGG |
| *sXbp1* | CTGAGTCCGAATCAGGTGCAG | GTCCATGGGAAGATGTTCTGG |
| *uXbp1* | TGGCCGGGTCTGCTGAGTCCG | GTCCATGGGAAGATGTTCTGG |
| *gpx1* | AATGTCGCGTCTCTCTGAGG | TCCGAACTGATTGCACGGG |
| *gpx4* | GCAGGAGCCAGGAAGTAAT | GGCTGGACTTTCATCCATTT |
| *atg12* | TGCTGAAGGCTGTAGGAGACACT | GATGAAGTCAATGAGTCCTTGGA |

**SUPPLEMENTARY FIGURE LEGENDS**

**Supplementary Figure 1.** (**A**) Protein levels of pIRE1α, IRE1α, BiP, sXBP1, and uXBP1 were determined in untreated *Xbp1****^f/f^*** and *Xbp1****^Δhepa^*** mice. GAPDH was used as a loading control. Relative protein levels were quantified using ImageJ software. Data are expressed as mean ± SEM (^#^p<0.05^,##^p<0.01;^#^ intergroup; *N* = 5-7 per experimental group). (**B-D**) *Xbp1^f/f^* and *Xbp1^∆hepa^* mice were intraperitoneally injected with APAP [500mg/kg] challenge, and sacrificed 24h later. Markers of liver damage were assessed. Serum ALT (**B**), AST (**C**) and LDH (**D**) levels are represented as U/L. (**E**) Liver pictures in *Xbp1****^f/f^*** and *Xbp1****^Δhepa^*** mice were taken. Scale bars, 1cm. (**F**) Protein levels of cleaved caspase-3 (CC3), CYP2E1 and ZO-1 of 24h after high dose APAP were determined by Western Blot. Relative protein levels were quantified using ImageJ software. Data are expressed as mean ± SEM (*p<0.05-****p<0.0001;^#^p<0.05-^###^p<0.0001;* intragroup,^#^ intergroup; *N* = 5-7 per experimental group).

**Supplementary Figure 2**. *Xbp1^f/f^* and *Xbp1^∆hepa^* mice were challenged with APAP [500mg/kg] at early (1-2h) stages of hyperacute liver injury. Control mice were injected with an equal volume of PBS with 10% DMSO. (**A**) Representative H&E staining was performed in paraffin liver sections of DMSO-treated *Xbp1^f/f^* and *Xbp1^∆hepa^* animals. Scale bars, 100μm. (**B**) Representative TUNEL staining performed on frozen liver sections. Scale bars, 100μm. (**C**) Representative IHC staining for CYP2E1 for the same livers and Scale bars, 100μm. (**D**) TUNEL-positive cells per view field were quantified. (**E**) Quantification of CYP2E1-positive area per view field.

**Supplementary Figure 3. Ablation of XBP1 reduced oxidative stress and lipid accumulation in the liver.** (**A**) Representative 4-HNE staining performed on paraffin liver sections and quantification of positive cells per view field. Scale bars, 100μm. (**B**) Representative ORO staining performed on frozen liver sections and quantification of ORO positive cells per view field. Scale bars, 100μm. (**C**) Liver triglycerides content was tested by enzymatic colorimetric tests. Scale bars, 50μm. (**D**) Serum triglycerides level represented as mg/dl. (**E**) mRNA level of *Ho-1* was determined by RT-qPCR. Data are expressed as mean ± SEM and graphed (*p<0.05, ***p<0.001, ****p<0.01, ^##^p<0.01, ^####^p<0.0001; * intragroup; ^#^ intergroup; *N* = 3-5 per experimental group).

**Supplementary Figure 4. Mice with hepatocyte-specific deletion of XBP1 display decreased inflammatory response 24h after APAP challenge.** (**A**) Representative CD45 staining performed on frozen liver sections and quantification of CD45 positive cells per view field. Scale bars, 100μm. (**B**) Representative CD11b staining performed on frozen liver sections and quantification of CD11b positive cells per view field. Scale bars, 100μm. (**C**) Representative F4/80 staining performed on frozen liver sections. Scale bars, 100μm. (**D**) Quantification of CD45+, CD11b+ and F4/80+ positive cells per view field. (**E**) mRNA level of *Tnfα, IL-1β* and *Nrlp3* were determined by RT-qPCR. Data are expressed as mean ± SEM and graphed (*N* = 3-7 per experimental group; *p<0.05-****p<0.0001, ^#^p<0.05-^###^p<0.0001; *intragroup, ^#^intergroup; *N* = 3-7 per experimental group). Arrows denote positive cells.

**Supplementary Figure 5**. Transmission electron microscopy (TEM) images were obtained from *Xbp1^f/f^* and *Xbp1^∆hepa^ animals*, 24 h after treatment with vehicle and APAP. N = nucleus, M = mitochondria, A = autophagosome, L = lipid, ER = endoplasmic reticulum.
