# Supplementary figures and images for: Genetic and pharmacological inhibition of XBP1 protects against APAP hepatotoxicity through the activation of autophagy
Source: Cell Death Dis. 2022 Feb 10;13(2):143. doi: 10.1038/s41419-022-04580-8 (PMC8831621; doi:10.1038/s41419-022-04580-8)

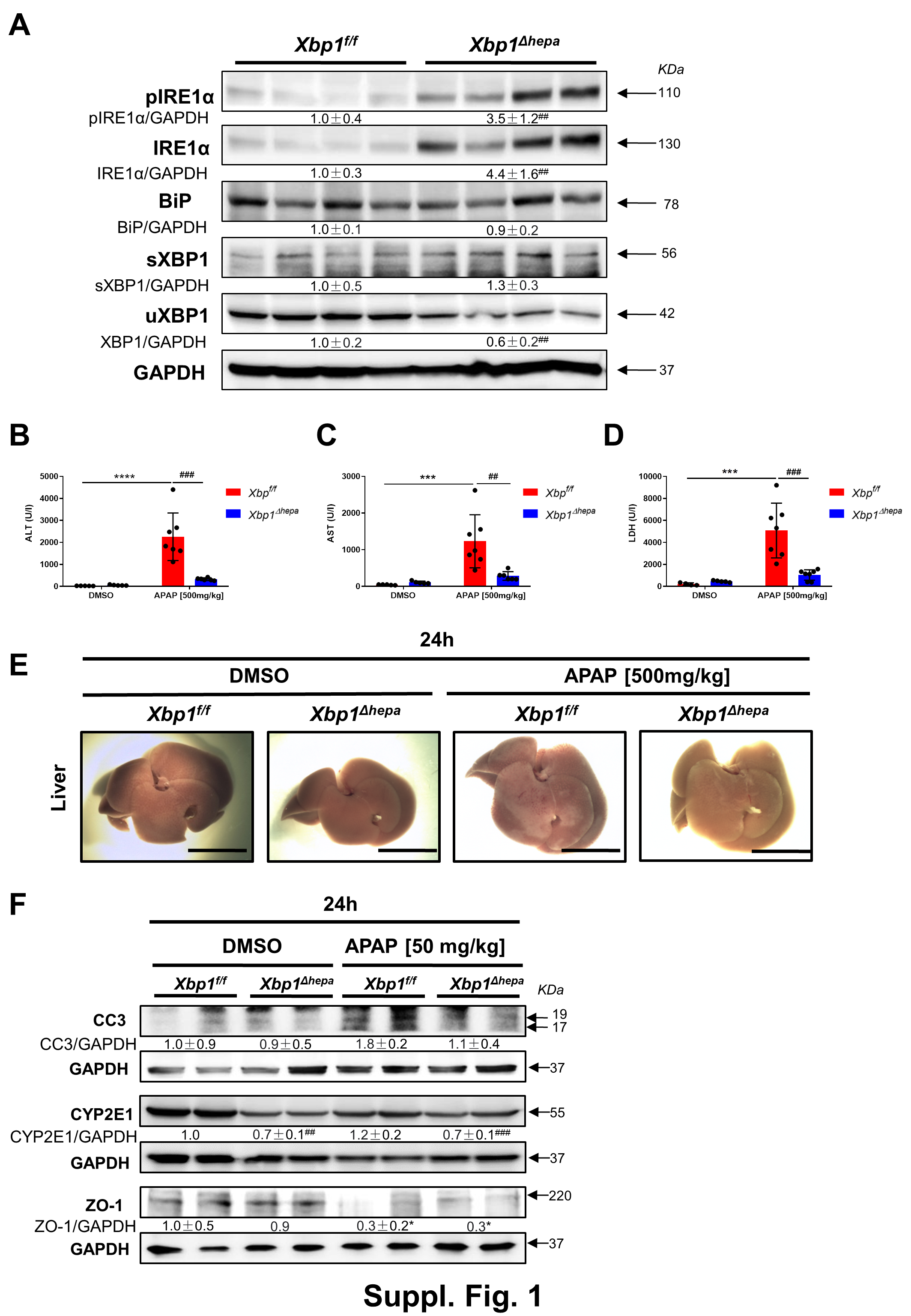

Supplement: Supplementary file 2 — Suppl. Fig. 1 [file 41419_2022_4580_MOESM2_ESM.tif]

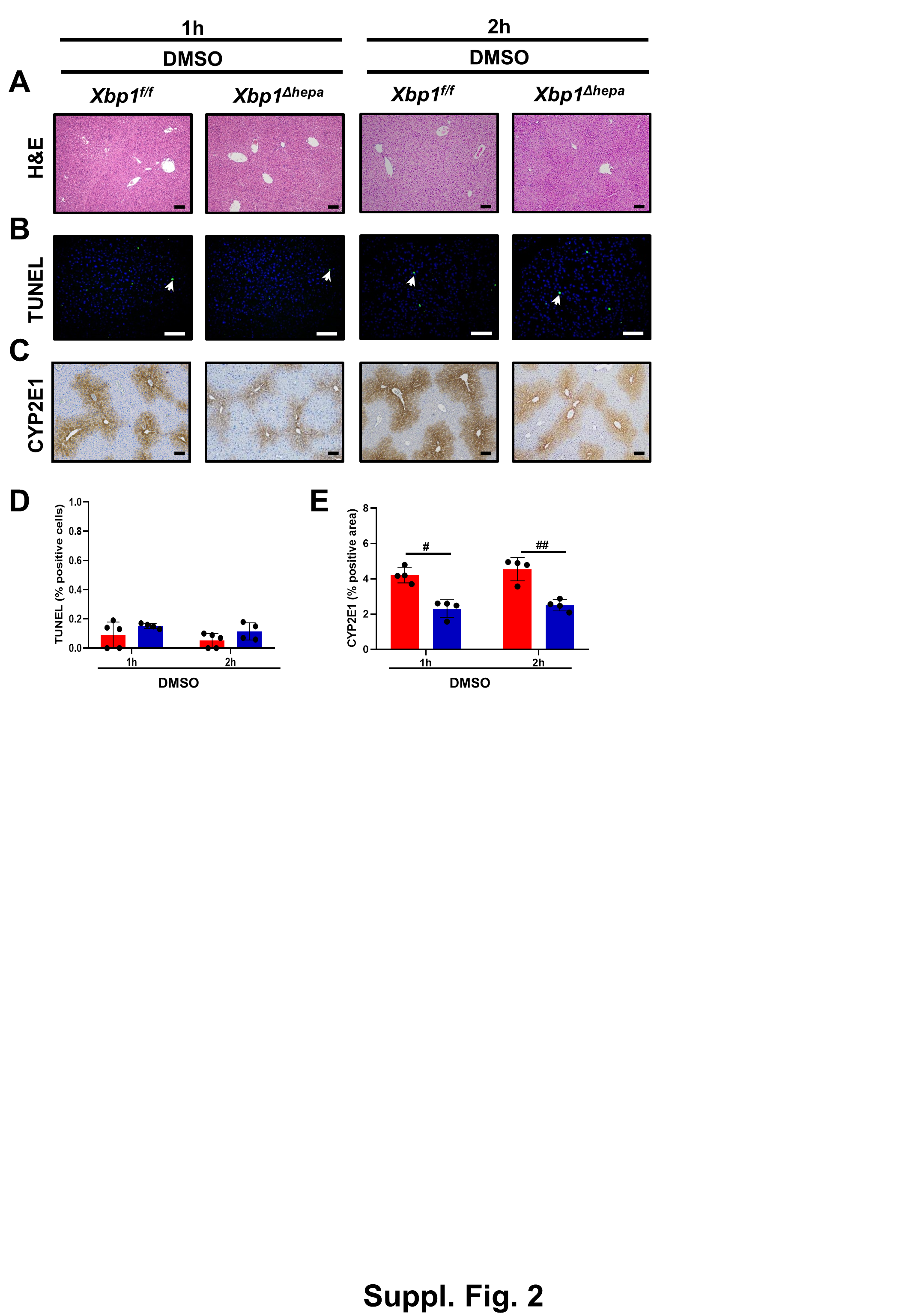

Supplement: Supplementary file 3 — Suppl. Fig. 2 [file 41419_2022_4580_MOESM3_ESM.tif]

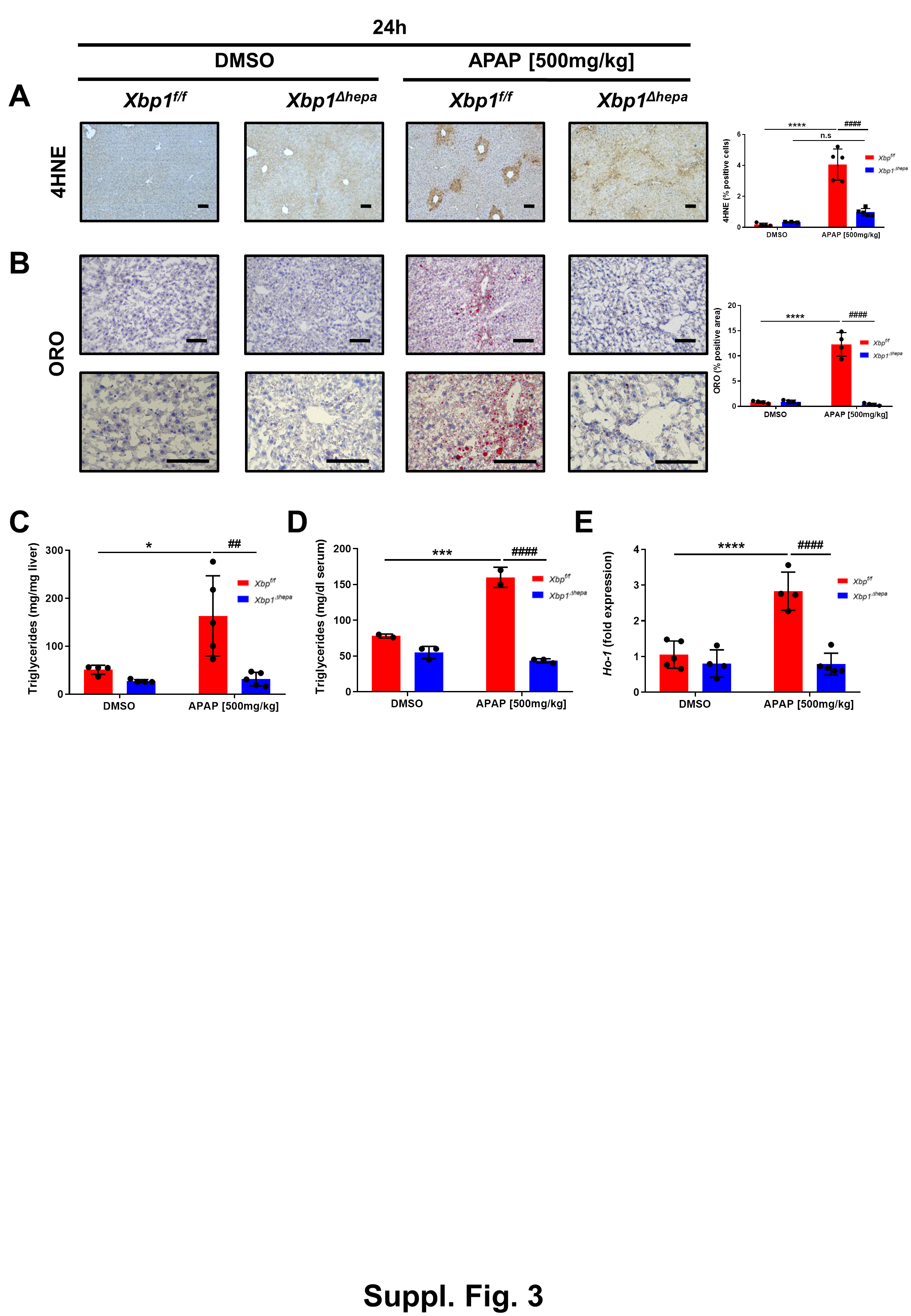

Supplement: Supplementary file 4 — Suppl. Fig. 3 [file 41419_2022_4580_MOESM4_ESM.tif]

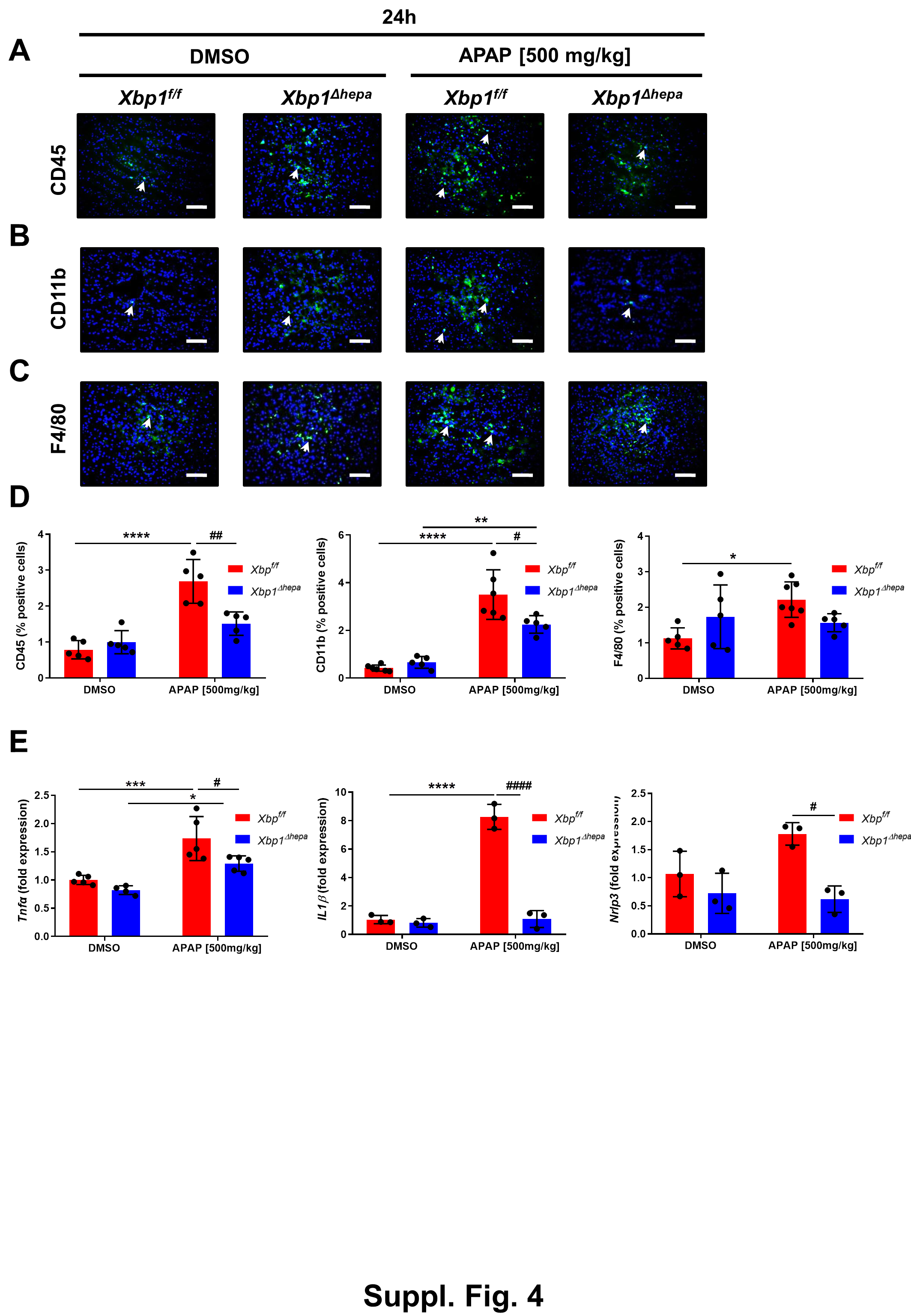

Supplement: Supplementary file 5 — Suppl. Fig. 4 [file 41419_2022_4580_MOESM5_ESM.tif]

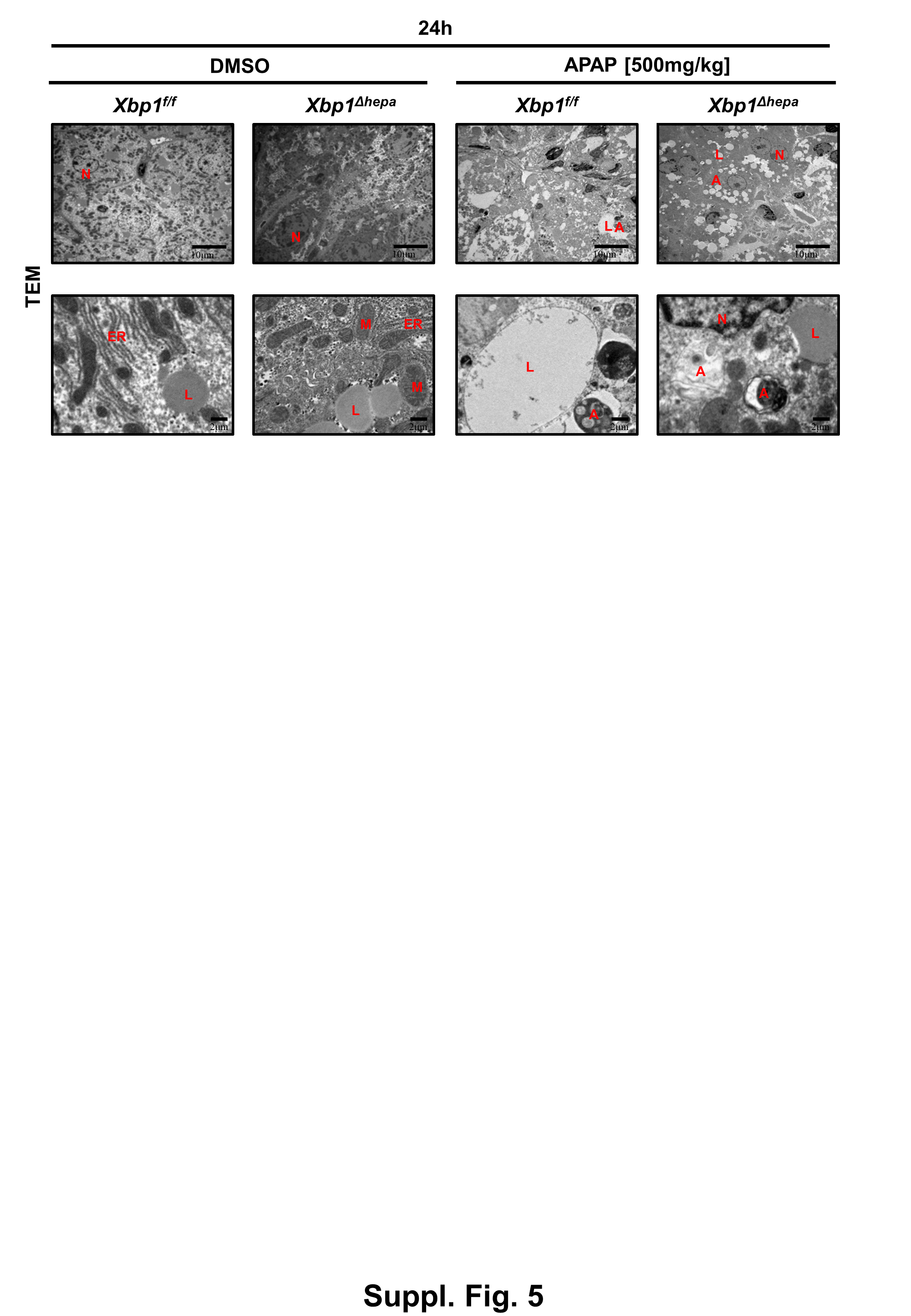

Supplement: Supplementary file 6 — Suppl. Fig. 5 [file 41419_2022_4580_MOESM6_ESM.tif]
